# A Lecture on the Frequent Repetition of Doses

**Published:** 1883-03

**Authors:** A. A. Smith

**Affiliations:** Professor of Materia Medica and Therapeutics, and of Clinical Medicine


A LECTURE ON THE FREQUENT REPETITION OF DOSES. DELIVERED AT THE BELLEVUE HOSPITAL MEDICAL COLLEGE, by A. A. SMITH, M. D., Professor of Materia Medica and Therapeutics, and of Clinical Medicine.

GENTLEMEN: I propose to direct your attention this morning to the subject referred to at my last lecture, namely, the frequent repetition of doses. This subject is a very important one, and one regarding which it is very difficult to establish any arbitrary rules. In the case of chronic diseases, where it is necessary to continue the treatment for a long time, the plan of administering the medicine in larger doses at intervals of five or six hours is probably the best one which can be adopted. For example, if you were prescribing some preparation of iron in a case of anæmia, it would be unnecessary to give it oftener than three times daily. Again, in certain cases it may be desirable to produce the full effect of the drug at a single dose, as in the administration of a cathartic, or of quinine to reduce temperature.

In other cases, however, it is desired, in administering medicinal remedies, to keep up their continued effect, and the question arises, whether we can accomplish this purpose better by giving them in smaller doses at frequent intervals than by giving them in large doses at much longer intervals, the total amount of the drug in the end being, perhaps, the same in either case. It is a fact with which you are acquainted that certain drugs become absorbed and produce their effect upon the system in a very short time, and they may also be eliminated very rapidly, while others act slowly and are eliminated after a longer interval.

It is not my intention this morning to deliver a scientific lecture; I shall make certain statements based upon clinical facts for which I shall not attempt to give any explanation.

The first drug to which I would call your attention in connection with the subject of the lecture is the chlorate of potash. It may not be unknown to most of you that this drug has at times been administered in sufficiently large doses to produce a dangerous inflammation of the kidneys. Special attention has been called to this fact by Dr. Jacobi, of this city, and also by other authors. This danger can be avoided by administering the drug in small doses frequently repeated. In writing the prescription, a teaspoonful of the solution may be made to represent as much of the drug as you wish to give; or, if it be in a more concentrated form, the patient may add water to it. Grain doses given every half-hour in scarlet fever, diphtheria, tonsillitis, etc., will produce the same results as larger doses, without the danger of the evil effects resulting from the accumulation of the drug in the system, as sometimes happens when it is administered in the ordinary way. Indeed, I believe they will produce better results upon the throat inflammations.

For the treatment of neuralgia, croton chloral has for a long time been given in large doses, as from five to eight grains, repeated every two hours, until fifteen grains are taken. But allow me to suggest what I consider a better mode of administering the drug—that is, to give a grain of it, prepared as you please, either in liquid or pill form, every half hour until the neuralgic symptoms are relieved. A solution of which a teaspoonful represents a grain of the croton chloral may be made, having scarcely any of the bad taste which usually belongs to this medicine when given in large doses. I may here remark that one of the important advantages connected with the frequent repetition of doses is the fact that the medicine may be so largely diluted with water or other vehicle as to be rendered comparatively tasteless, and harmless to the mucous membrane of the stomach.

You will often be called upon to treat very obstinate cases of urticaria, and you will be put to your wits' end to know what to do. The plan ordinarily suggested is to give alkalies, as the bicarbonate of sodium, or magnesium; but, if you will give the patient two grains of the salicylate of sodium every hour or halfhour, you will usually be enabled to effect a cure even in obstinate cases, except those of a chronic nature. Two grains of the salicylate of sodium administered in a teaspoonful of water is almost tasteless, and may be given without producing disturbance of digestion. Urticaria is often caused by the administration of full doses of balsam of copaiba in cases of urethritis, or inflammation of other mucous membranes, and it may seem strange to you when I make the statement that a single drop of the same drug given every half hour will sometimes control urticaria. I have no explanation to offer, but I make the statement not alone upon the authority of others; I myself have often observed the efficacy of the treatment, although not so frequently as in the treatment by the salicylate of sodium.

Fowler's solution, or the liquor potassii arsenitis, half a drop given every half-hour for six or eight doses, will often relieve the vomiting which occurs after a debauch. It will also relieve the morning vomiting of drunkards, and is of decided benefit in the sympathetic nausea and vomiting of pregnancy.

Jaborandi has been given in large doses with a view to exciting perspiration in cases of Bright's disease, but the very serious objection has been found to its administration in this manner, that it sometimes has a very depressing effect upon the heart's action, resulting in some cases fatally. Now, five to ten-minim doses of the fluid extract of jaborandi given every hour or half-hour will produce marked perspiration without causing any unpleasant effects upon the heart. I sometimes combine with the jaborandi the tincture of digitalis, with a view to counteract any possible evil influence which the former drug may have upon the heart. So dangerous do I consider large doses of jaborandi that I often hesitate long before administering it, especially in the uræmia of the puerperal state.

You will please remember that the amount of the medicines administered is not so small as you may at first suppose, especially if you take into consideration their strength and the frequency of their repetition.

The next preparation of which I shall speak is a solution of the sulphate of atropine, one one-hundredth of a grain in a goblet of water, a teaspoonful of which shall constitute a dose, amounting in all to about sixty doses. Now, you will often be called to see cases of supposed croup, but whieh, in the majority of instances, prove to be cases of false croup of a reflex origin. Ordinarily, you will be able to relieve these patients by giving them a teaspoonful of this preparation every hour. It is possible the remedy acts slightly as a stimulant of the respiratory center; it is also possible that it has some influence upon muscular contraction or relaxation; at all events, clinical experience proves that it is of benefit in these cases. The dose may be repeated every hour or half-hour, according to the severity of the attack. If the child's face begins to flush and show signs of the physiological effects of the drug, the dose can be reduced in frequency. It should be remembered that when thus administered the equivalent of a full dose of the drug will soon be reached. Do not forget in these cases to give an emetic if there is anything in the stomach which may be causing the spasm, or a cathartic if there be reason to suspect intestinal disturbance as the cause.

The bromides are largely used in the treatment of the nervous and febrile disturbances of children, but an objection to them is the fact that the little patients do not take them readily, because of the taste; the bromide of sodium is, perhaps, as little disagreeable as any of the preparations. This objection can be avoided by giving small doses frequently repeated; for instance, a few grains dissolved in half a tumblerful of water, a teaspoonful representing a half grain, or a grain even, administered every ten or fifteen minutes. When given in this manner, the bromides often prove of great benefit in the nervous disturbances arising from dentition and other causes, and in relieving the fever which, in children, usually attends a slight degree of excitement of any kind. I have seen an elevation of the temperature in children where it could not be traced to any other cause than the excitement incident to their afternoon play. A temperature which might indicate a sickness of considerable gravity in the adult, if it occur in a child may be of comparatively little importance. In such cases the bromides, administered in small doses, say a grain or two at intervals of ten or fifteen minutes, will often prove of great benefit.

I began the use of some of these remedies administered in this manner on the recommendation of others, and I must say in a somewhat skeptical frame of mind, thinking that the effect which they produced was probably due to the moral influence upon the patient, or that it had no foundation in fact, it being a mere coincidence that the drugs were administered at a time when the patients would have recovered in the absence of any treatment; but, having seen benefit follow their administration repeatedly, I concluded they must have a wider range of usefulness, and began to use them much more frequently.

You will often meet with children of a nervous, excitable frame of mind, who are, perhaps, naturally of a sensitive, nervous temperament, who are disturbed by the slightest noise, and are unable to go to sleep before ten or eleven o'clock at night. In such cases you will find it necessary to give a nervous sedative. An excellent effect will be produced by chamomilla in some one of its forms, as the tincture, administered in minim doses, every fifteen or twenty minutes. It is tonic as well as sedative. It is a better sedative in such cases than the hydrate of chloral, which is liable to affect the digestion. It is harmless when given in larger doses. Put a teaspoonful into a half-tumblerful of water, and let the child drink it freely.

One of the most important remedies which can be administered with great benefit in frequently repeated doses is ipecac. You are aware that a teaspoonful of the syrup of ipecac is likely to produce emesis; but it is also a fact, regarding which I was at first quite skeptical, that a single drop of the wine of ipecac will often arrest obstinate vomiting. It should be repeated every ten or fifteen minutes. When administered in this manner, I have often known it to relieve vomiting from different causes, among which are pregnancy and subacute gastritis. Children often vomit from very slight causes, and are liable to suffer from diarrhœa and vomiting which have no other assignable cause than disurbance of digestion. A single drop of the wine of ipecac, repeated every fifteen or twenty minutes, will often produce the most marked relief, both from the vomiting and from the diarrhœa. Administered in this manner, the drug is not nauseous, and is easily taken.

I now make a statement, upon the authority of Trousseau and his enthusiastic successor, which may appear to you, as it once did to me, incredible—viz., that one sixtieth of a grain of calomel taken every hour for ten or twelve hours will relieve the headache of syphilis occurring at night. I have administered it in one-fortieth grain doses in this manner and have obtained the results which they claimed for it, but I have not yet tried it in sixtieth-grain doses. The relief was very marked by the second or third night. It is not intended to take the place of iodides which are given in such cases. Doubtless the calomel, when administered in such small doses, is all taken up into the system.

Nursing children often vomit or regurgitate their food; this has been relieved repeatedly in my experience by giving them a teaspoonful of a solution of one grain of calomel to the pint of water every ten or fifteen minutes. In order to dissolve it, the calomel should first be put into an ounce of lime-water, and then into the pint of pure water. One twenty-fourth of a grain of mercury with chalk, administered every fifteen or twenty minutes, is often of great benefit in the vomiting and non-inflammatory diarrhœa of children. Where the diarrhœa is accompanied by mucous passages, indicative of a certain degree of inflammatory action, or enteritis, benefit will be derived from the administration of one teaspoonful of a solution of bichloride of mercury (corrosive sublimate), one grain to the quart, every hour. The dose may seem very small, but it must be remembered that the dose for an adult is only one sixtieth to one thirtieth of a grain, and, when administered in this manner, the full dose for a child is reached within a few hours.

Another extraordinary statement, which at first seemed to me to be fabulous, and may seem so to you, but which, nevertheless, you will find to be based upon clinical facts: Put a grain of tartar emetic into one quart of water; teaspoonful doses of this solution every half-hour will prove effectual for the relief of the wheezing and cough accompanying a slight bronchitis in children.

A single drop of the tincture of nux vomica given every ten minutes will often produce most marked relief in sick headache not of a neurotic origin. It should be given immediately after or soon after meals.

It is well known that cantharides, when given in large doses, is liable to cause inflammation of the urinary tract; but it has been found that a single drop of the tincture every hour will in many cases relieve vesical catarrh.

You probably have heard that digitalis has been used in cardiac disease. Certainly if you have not heard of it you will, and, if you have already heard of it, you will hear of it again. particularly at the clinics. Ordinarily, it is administered in considerable doses only three or four times a day; but I do not hesitate to say that the frequent repetition of small doses will produce much more benefit than larger doses at longer intervals. A single drop of the tincture of digitalis, given to a patient suffering from symptoms due to organic heart disease when digitalis is indicated, administered at intervals of an hour or half-hour, according to the severity of the symptoms, will often give greater relief than larger doses, and without liability to ill effects.

For the diarrhœa of children, accompanied with slight inflammation, straining, and the passage of jelly-looking matter, but not true dysentery, five drops of castor-oil, given every hour in water with sugar and gum, is an excellent remedy.

A gentleman in this city, of authority in the specialty of venereal diseases, says he has given greater relief in a short time, in cases of orchitis and epididymitis, by the administration of two-minim doses of the tincture of pulsatilla every hour than by any other mode of treatment. I can testify to the great benefit derived from the drug administered in this manner in dysmenorrhœa not of a membranous, obstructive, or neuralgic character.

One of the most distressing symptoms from which many women suffer at the menopause is flatulence, and a sensation of fluttering or palpitation at the pit of the stomach, an effectual remedy against which is the extract of calabar bean in one-fiftieth-grain doses, repeated every half-hour for six or eight doses. It may be repeated in the same way after stopping it for three hours.

In cases of amenorrhœa not dependent upon anæmia, benefit may be derived from minim doses of the fluid extract of ergot administered every half-hour for five or six hours the day before the flow should begin, and again on the day on which it should occur. Contradictory as it may seem, when administered in the same manner the fluid extract of ergot is of benefit in cases of excessive menstruation.

Aconite is one of the drugs to which you will probably have occasion to resort frequently when you enter upon the active practice of medicine. It has for a long time been used in quite small doses, but not so frequently repeated as it might be with benefit. There are many cases of febrile movement, with dry, hot skin, a full, bounding pulse, the mucous membrane of the throat and nose probably dry—cases in which the febrile movement is not the commencement of one of the continued fevers; the tincture of aconite, one third to one half a minim given every fifteen minutes, will be found of decided benefit. Visiting the patient shortly after the commencement of this treatment, you will often find him in a little perspiration; the medicine may then be administered at longer intervals, every half-hour or longer, according to the indications. The tincture of aconite, administered in a similar manner, is also useful in cases of commencing so-called cold in the head. It is likewise useful in cardiac hypertrophy with palpitation, severe headache, and disturbances of the nervous system due to increased force of the heart-beat.

Two minims of the tincture of hamamelis every half-hour will often control hæmorrhages. I was at first inclined to look upon this statement with a great deal of distrust, but I have since tried it in cases of hæmorrhage from the nose, from the uterus, and in the hæmorrhage from hæmorrhoids, and have found it of great benefit.

The tincture of belladonna in minim doses, given every half-hour, is a good remedy in cases of nasal catarrh, and bronchitis accompanied by free secretion. You should cease to give the drug for a while after eight or ten doses have been administered, as it is less quickly eliminated from the system than the other medicines of which we have already spoken. In cases of pulmonary œdema with failure of heart power, belladonna thus administered is of benefit in retarding the exudation of serum, and in overcoming the failure of heart power.

Two grains of the chloride of ammonium, combined with ten or fifteen minims of the tincture of cubebs, given every half-hour, oftentimes controls acute pharyngitis and superficial inflammations of the other tissues about the throat. For inflammation of the throat dependent upon a gouty diathesis, add to this mixture ten minims of the ammoniated tincture of guaiac, and administer every hour.

In the headache of migraine, one grain of the citrate of caffeine given every half-hour will often produce most marked relief.

In neuralgias about the face or head, three-minim doses of the tincture of gelseminum every half-hour will often act almost miraculously and leave no ill effects.

For certain kinds of headaches (especially those which are periodical and not of malarial origin), fifteen-minim doses of fluid extract of guarana given every fifteen minutes will very frequently relieve. If it does not relieve in four doses, increase the dose to thirty minims.—*New York Medical Journal.*

